# Non-natural Aldol Reactions Enable the Design and Construction of Novel One-Carbon Assimilation Pathways *in vitro*

**DOI:** 10.3389/fmicb.2021.677596

**Published:** 2021-06-02

**Authors:** Yufeng Mao, Qianqian Yuan, Xue Yang, Pi Liu, Ying Cheng, Jiahao Luo, Huanhuan Liu, Yonghong Yao, Hongbing Sun, Tao Cai, Hongwu Ma

**Affiliations:** ^1^Biodesign Center, Key Laboratory of Systems Microbial Biotechnology, Tianjin Institute of Industrial Biotechnology, Chinese Academy of Sciences, Tianjin, China; ^2^Tianjin Institute of Industrial Biotechnology, Chinese Academy of Sciences, Tianjin, China; ^3^State Key Laboratory of Food Nutrition and Safety, Tianjin University of Science and Technology, Tianjin, China; ^4^Key Laboratory of Systems Bioengineering (Ministry of Education), SynBio Research Platform, Collaborative Innovation Center of Chemical Science and Engineering (Tianjin), School of Chemical Engineering and Technology, Tianjin University, Tianjin, China

**Keywords:** synthetic methylotrophy, computational pathway design, allose 6-phosphate, *In vitro* pathway construction, aldolase reaction, glycolaldehyde-allose 6-phosphate assimilation pathway

## Abstract

Methylotrophs utilizes cheap, abundant one-carbon compounds, offering a promising green, sustainable and economical alternative to current sugar-based biomanufacturing. However, natural one-carbon assimilation pathways come with many disadvantages, such as complicated reaction steps, the need for additional energy and/or reducing power, or loss of CO_2_, resulting in unsatisfactory biomanufacturing performance. Here, we predicted eight simple, novel and carbon-conserving formaldehyde (FALD) assimilation pathways based on the extended metabolic network with non-natural aldol reactions using the comb-flux balance analysis (FBA) algorithm. Three of these pathways were found to be independent of energy/reducing equivalents, and thus chosen for further experimental verification. Then, two novel aldol reactions, condensing D-erythrose 4-phosphate and glycolaldehyde (GALD) into 2*R*,3*R*-stereo allose 6-phosphate by DeoC or 2*S*,3*R*-stereo altrose 6-phosphate by TalB^F178Y^/Fsa, were identified for the first time. Finally, a novel FALD assimilation pathway proceeding *via* allose 6-phosphate, named as the glycolaldehyde-allose 6-phosphate assimilation (GAPA) pathway, was constructed *in vitro* with a high carbon yield of 94%. This work provides an elegant paradigm for systematic design of one-carbon assimilation pathways based on artificial aldolase (ALS) reactions, which could also be feasibly adapted for the mining of other metabolic pathways.

## Introduction

**Figure 1:**
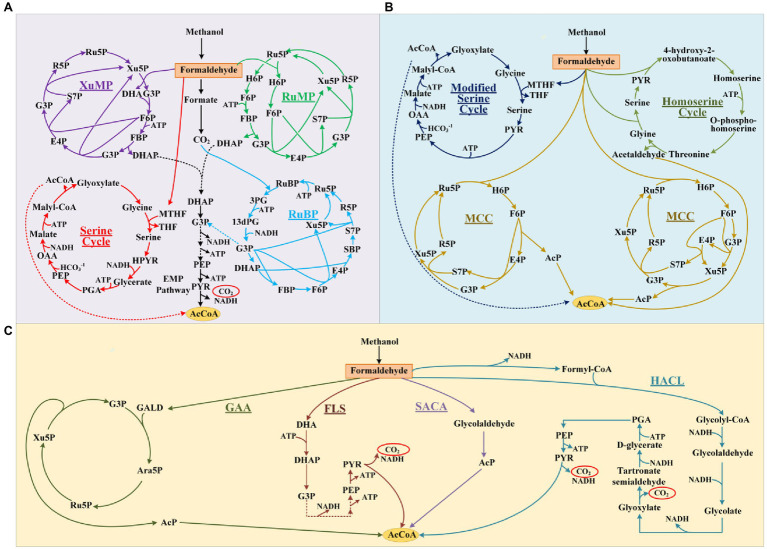
Methanol assimilation pathways. The natural pathways **(A)** and artificial pathways without non-natural reactions **(B)** or artificial pathways based on non-natural reactions **(C)**. R5P, D-ribose 5-phosphate; Ru5P, D-ribulose 5-phosphate; Xu5P, D-xylulose 5-phosphate; DHA, dihydroxyacetone; G3P, D-glyceraldehyde 3-phosphate; F6P, D-fructose 6-phosphate; FBP, fructose 1,6-bisphosphate; E4P, D-erythrose 4-phosphate; H6P, D-hexulose 6-phosphate; S7P, D-sedoheptulose 7-phosphate; SBP, D-sedoheptulose 1,7-bisphosphate; DHAP, dihydroxyacetone phosphate; PEP, phosphoenolpyruvate; PYR, pyruvate; RuBP, D-ribulose 1,5-bisphosphate; 3PG, D-glycerate 3-phosphosphate; 13dPG, 3-phospho-D-glyceroyl phosphate; OAA, oxaloacetate; GALD, glycolaldehyde; Ara5P, D-arabinose 5-phosphate; PGA, D-glycerate 2-phosphate; AcP, acetyl-phosphate; and AcCoA, acetyl-coenzyme A.

Growing concerns over global fossil-resources and food shortages have motivated the development of sustainable commodity biomanufacturing from alternative resources (Clomburg et al., 2017). Over the past decade, advances in the bioconversion of non-food, low-cost, and abundant one-carbon compounds such as methanol, formate, and CO_2_ using native or synthetic methylotrophs highlighted a potentially green and economical alternative to current sugar-based biomanufacturing (Liang et al., 2020; Mao et al., 2020; Nguyen and Lee, 2020). Notably, electron-enriched methanol (CH_4_O) is expected to support more economical biosynthesis of chemicals with higher theoretical carbon-molar yields than sugars. However, the development of efficient methylotrophs, especially ones with carbon-conserving metabolism, is still hindered by the inherent drawbacks of natural methanol assimilation pathways (Figure 1A). These include complicated pathway, high energy, and reducing force requirements, as well as carbon loss during the conversion of methanol into the key metabolite acetyl-coenzyme A (AcCoA; Yang et al., 2019b; He et al., 2020).

Substantial efforts have been devoted to designing artificial methanol assimilation pathways that can outperform their natural counterparts (Figure 1B). Yu and Liao (2018) simplified the natural serine cycle by assimilating formaldehyde (FALD) instead of formate and halving the number of steps from serine to phosphoenolpyruvate. He et al. (2020) proposed the homoserine cycle, which halves the number of required enzymes and quarters the ATP demand for AcCoA synthesis compared with the serine cycle. However, both these pathways are still ATP- and NADH-dependent, which not only necessitates efficient cofactor regeneration but also lowers the yields of AcCoA derivatives (Figure 1B). By combining the ribulose monophosphate (RuMP) pathway and non-oxidative glycolysis (NOG), Bogorad et al. (2014) designed a carbon-conserving ATP-independent methanol condensation cycle (MCC), which achieved a final carbon yield of 80% for the synthesis of AcCoA derivatives *in vitro*. Although its yield surpassed the 67% theoretical yield of native RuMP and XuMP pathways coupled with the EMP, the construction of MCC pathway is still complicated, because it requires nine enzymes (Yang et al., 2019b).

Most methanol assimilation pathways must form FALD for further metabolism, making FALD assimilation a key step in methanol metabolism (Müller et al., 2015; Whitaker et al., 2017; Meyer et al., 2018). The introduction of computationally designed formolase (Fls; Siegel et al., 2015) or evolved glycolaldehyde synthase (Gals; Lu et al., 2019), which can condense three or two FALD molecules into dihydroxyacetone (DHA) or glycolaldehyde (GALD), enabled the construction of novel FALD assimilation pathways. However, the Fls-based pathways still exhibit one-third carbon loss during AcCoA synthesis. Our colleagues proposed a Gals-based pathway named synthetic acetyl-CoA (SACA), in which GALD is converted into acetyl-phosphate (AcP) by a repurposed PK (Lu et al., 2019). However, the *in vitro* performance of this pathway was not satisfactory, with a final carbon yield of ~50%, probably due to the unfavorable substrate affinity and low catalytic efficiency of Gals. Chou et al. (2019) constructed a kinetically favorable pathway for the bioconversion of FALD into GALD by coupling an evolved 2-hydroxyacyl-CoA lyase (Hacl) with acyl-CoA reductase (Acr; Figure 1C), which finally achieved an 84% carbon yield for glycolate production *in vivo*. Although the current route from glycolate to AcCoA is still long and subject to carbon loss (Figure 1C), this kinetically favorable GALD pathway laid the foundation for prospectively more efficient bioconversion of GALD into AcCoA by coupling it with other artificial routes.

Although pathway design based on artificial/non-natural reactions has enabled the construction of brand-new methanol assimilation pathways that avoid the inherent drawbacks of their natural counterparts, rich experience is indispensable for assembling artificial reactions into pathways. Model-based pathway design using mathematical algorithms is increasingly favored for novel one-carbon assimilation pathway mining due to their systematic and innovative advantages (Trudeau et al., 2018; Yang et al., 2019b). Previously, our group constructed a large metabolic network model (Yang et al., 2019b) by integrating 6,578 known reactions from MetaCyc (Caspi et al., 2018) and 73 hypothetical aldolase (ALS) reactions from ATLAS (Hadadi et al., 2016; Hafner et al., 2020). By conducting comb-FBA, we predicted 59 ATP/NAD(P)H independent FALD assimilation pathways with 100% theoretical carbon yield for AcCoA-derived acetate production. Finally, the glycolaldehyde assimilation (GAA) pathway (Figure 1C) was constructed and verified with a high acetate yield of 88% *in vitro*. Inspired by this result, here, we further artificially proposed 28 non-natural aldolase reactions based on the aldol reaction mechanism, which were not present in the ATLAS database. These 28 possible aldolase reactions were added into an extended known reaction set for pathway calculation using our previously developed comb-FBA algorithm. Eight novel carbon conserving FALD assimilation cycles were calculated. Two novel aldol reactions were identified with feasible aldolases. Finally, a novel FALD assimilation pathway, proceeding *via* the condensation of GALD and E4P into allose 6-phosphate, was named as the glycolaldehyde-allose 6-phosphate assimilation (GAPA) pathway and constructed *in vitro*.

## Materials and Methods

### Metabolic Reaction Set for Pathways Design

The reactions set^6578^ constructed in our previous work (Yang et al., 2019b), containing 6,566 unblocked MetaCyc reactions, 11 exchange reactions and one objective reaction (Supplementary Material; Supplementary Table A) was used as the base reaction set. Then, a total of 28 newly proposed aldol reactions using FALD/GALD as acceptor or donor, and theoretically feasible based on the aldolase reaction mechanism (Supplementary Table 1), were added to this base set for pathway calculation.

### Calculation Method

The parsimonious flux balance analysis (pFBA) algorithm, which minimizes the sum of flux distribution, was used to obtain the solution (Lewis et al., 2010). The comb-FBA (combination of combinatorial algorithm and pFBA algorithm) algorithm developed in our previous work (Yang et al., 2019b) was used for pathway design. Simulations were performed in Python using the COBRApy (Ebrahim et al., 2013). The exchange reaction for acetate was defined as the objective reaction and the input rate of FALD was set to 10 mmol·(g DCW)^−1^·h^−1^.

### Reagents, Strains and Media

D-Erythrose 4-phosphate (E4P), glycolaldehyde, D-glucose 6-phosphate, D-mannose 6-phosphate, methoxyamine hydrochloride, pyridine and N-methyl-N-(trimethylsilyl) trifluoroacetamide (MSTFA) were purchased from Sigma-Aldrich (St. Louis, MO, United States). D-Allose and D-altrose were purchased from Macklin Biochemical (Shanghai, China). Other regents such as D-glucose, D-mannose, ATP, ADP, thiamine pyrophosphate (TPP), and MgCl_2_ were all purchased from Sangon Biotech (Shanghai, China), unless noted otherwise. *Escherichia coli* DH5α was used for plasmid construction and preservation. *Escherichia coli* BL21 (DE3) was used for protein expression. Luria-Bertani (LB) medium (10 g/L tryptone, 5 g/L yeast extract and 10 g/L NaCl) was used for *E. coli* cell culture and recombinant protein expression, supplemented with 50 μg/ml kanamycin or 100 μg/ml ampicillin when necessary.

### Construction of Plasmids

The plasmids used in this work are listed in Table 1. The *manA* gene was amplified by PCR from the genome of *E. coli* MG1655 using the primer pair manA-F/R (Table 2), and cloned between the *Nhe*I/*Xho*I restriction sites of pET28a(+), generating plasmid pET28a-*manA*. The plasmid pET28a-*alsE* was constructed analogously, using *Nhe*I and *Xho*I.

**Table 1:** Plasmids used in this study.

Plasmids	Relevant characteristics	NCBI-Protein ID	Ref.
pET32a-*talB^F178Y^*	Amp^R^, N-terminally His-tagged *talB^F178Y^*, inserted between the *BamH*I and *Xho*I sites	NP_414549	Yang et al., 2019b
pET28a-*fsa*	Kan^R^, N-terminally His-tagged *fsa*, inserted between the *Nhe*I and *Xho*I sites	NP_415346	Yang et al., 2019b
pET28a-*deoC*	Kan^R^, N-terminally His-tagged *deoC*, inserted between the *Nhe*I and *Xho*I sites	NP_418798	Yang et al., 2019b
pET28a-*rpiB*	Kan^R^, N-terminally His-tagged *rpiB*, inserted between the *Nhe*I and *Xho*I sites	NP_418514	Yang et al., 2019b
pET28a-*pgi*	Kan^R^, N-terminally His-tagged *pgi*, inserted between the *Nde*I and *EcoR*I sites	NP_418449	Chunling et al., 2017
pET28a-*manA*	Kan^R^, N-terminally His-tagged *manA*, inserted between the *Nhe*I and *Xho*I sites	NP_416130	This study
pET28a-*kdsD*	Kan^R^, N-terminally His-tagged *kdsD*, inserted between the *Nhe*I and *Xho*I sites	NP_417664	Yang et al., 2019b
pET28a-*alsE*	Kan^R^, N-terminally His-tagged *alsE*, inserted between *Nhe*I and *Xho*I sites	NP_418509	This study
pET28a-*fpk*	Kan^R^, N-terminally His-tagged *fpk*, inserted between the *Nhe*I and *Hind*III sites	BAF39468	Yang et al., 2019b

**Table 2:** Primers used in this study.

Primers	Sequence (5'-3')
manA-F	GTACGGCTAGCATGCAAAAACTCATTAACTC
manA-R	CATTGCTCGAGTTACAGCTTGTTGTAAACAC
alsE-F	TATCGGCTAGCATGAAAATCTCCCCCTCGTT
alsE-R	TGGTGCTCGAGTTATGCTGTTTTTGCATGAGG

### Protein Expression and Purification

*Escherichia coli* BL21 (DE3) strains carrying pET28a/pET32a-derived plasmids were used for protein expression. Cells were cultured in 5 ml of LB medium at 37°C and 220 rpm overnight. Then, 2 ml of the culture was used to inoculate 200 ml of LB medium in a 1 L shake flask, and grown at 37°C and 220 rpm. For induction of protein expression, isopropyl β-D-1-thiogalactopyranoside (IPTG) was added to a final concentration of 0.5 mM when the optical density at 600 nm (OD_600_) reached 0.6–0.8, and the cultivation temperature was set and maintained at 16°C for 16–18 h. Recombinant cells were harvested by centrifugation at 6,000 × *g* and 4°C for 40 min, and then re-suspended in 20 ml of phosphate buffer (PB, 50 mM, pH 7.5) containing 150 mM NaCl. The cell pellets were lysed using a high-pressure homogenizer at 4°C, and subsequently centrifuged at 6,000 × *g* and 4°C for 40 min to remove cell debris. The clear lysate was onto a Ni-NTA His-binding column and concentrated as described previously (Cui et al., 2018, 2019). The purity of the enzymes was analyzed by 12% SDS-PAGE (Supplementary Figure 1) and quantified using a bicinchoninic acid (BCA) Kit (CWBiotech, Beijing, China).

### Enzymatic Reaction Condition

For aldol reaction, the reaction systems containing 100 mM PB (pH = 7.0), 10 mM GALD, 2.5 mM E4P, 1 mM TPP, 5 mM MgCl_2_, and 5 mg/ml different aldolases were incubated at 37°C and 220 rpm for 2 h. For preparation of aldohexose 6-phosphate samples, the reaction systems containing 100 mM PB (pH = 7.0), 10 mM GALD, 2.5 mM E4P, 1 mM TPP, 5 mM MgCl_2_, and 5 mg/ml glucokinase (Glk) with 10 mM different aldohexoses (D-glucose, D-mannose, D-allose, or D-altrose) were incubated at 37°C and 220 rpm for 2 h. For isomerization of aldohexose 6-phosphate, the reaction systems containing 100 mM PB (pH = 7.0), 1 mM TPP, 5 mM MgCl_2_, 10 mM G6P, or M6P (or 10 mM ATP, 5 mg/ml Glk, and 10 mM allose/altrose) and 5 mg/ml different isomerases were incubated at 37°C and 220 rpm for 2 h. These reaction products were used for subsequently qualitative analysis.

### GC-TOFMS Analysis

Samples were dried using a CentriVap vacuum concentrator at 4°C. Then, the dried samples were dissolved in 50 μl of pyridine containing 40 mg/ml methoxyamine hydrochloride, and incubated for 90 min at 30°C. Finally, 50 μl of the MSTFA regent (containing 1% TMCS, v/v) was added to the sample aliquots, mixed well, incubated for 30 min at 37°C, and then sealed in amber gas chromatography-time of flight mass spectrometry (GC-TOFMS) sample vials containing glass inserts. The GC-TOFMS analysis was carried out on an Agilent 7890A gas chromatography system coupled with a quadrupole time-of-flight (Q-TOF) mass spectrometer and an inert electron ionization (EI) ion source (Agilent, United States). A DB-5MS capillary column coated with 5% diphenyl cross-linked with 95% dimethylpolysiloxane (30 m × 250 μm inner diameter, 0.25 μm film thickness; J&W Scientific, United States) was used. The oven temperature program was as follows: 60°C (initial), ramp to 180°C (2 min) at 10°C min^−1^, followed by a ramp to 230°C at 10°C min^−1^, then 5°C min^−1^ to 260°C, and finally 10°C min^−1^ to 320°C (4 min). The injection, transfer line, and ion source temperatures were 250, 290, and 230°C, respectively. The instrument was operated in electron impact mode at 70 eV. The sample injection volume was 1 μl with a split ratio of 10:1. Helium was used as the carrier gas, the front inlet purge flow was 3 ml min^−1^, and the gas flow rate through the column was 1.2 L min^−1^. The mass spectrometry data were acquired in full-scan mode in the m/z range of 35–650 at a rate of 5 spectra per second after a solvent delay of 7.5 min. Agilent MassHunter 10.0 software with NIST2020 libraries was used for data analysis.

### Measurement of Metabolite Concentrations

Acetyl-phosphate was converted into brown ferric acetyl-hydroxamate, and its concentration was analyzed by measuring the absorption at 505 nm (A_505_) using a multifunctional microplate reader (BioTek, Winooski, VT, United States) as previously reported (Bogorad et al., 2013). GALD was converted into a blue-violet diphenylamine derivative, and was analyzed by measuring the A_620_ as previously reported (Yang et al., 2019b). The reported values are the averages and SDs of three measurements.

## Results

### Proposal of Potential Aldolase Reactions With Different Product Stereoselectivity

**Figure 2:**
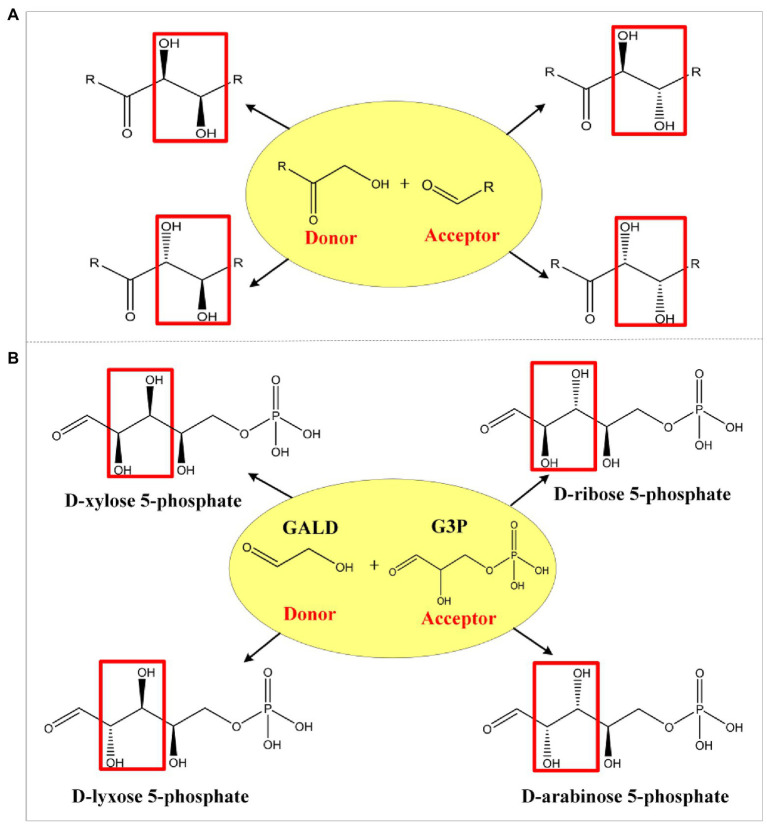
Schematic diagram of aldol reaction mechanism. Complementary stereochemistry of aldolases **(A)** and the four stereo configurations **(B)** when GALD serves as donor and G3P serves as acceptor.

Aldolases are proven tools for effective C-C bond formation with unrivaled efficiency in the synthesis of carbohydrates and complex polyhydroxylated molecules (Clapés et al., 2010; Windle et al., 2014; Roldán et al., 2017). Aldolases can generally use a broad range of aldehydes as acceptors, while some also showed an unprecedented donor spectrum, such as the transaldolase B mutant (TalB^F178Y^; Rale et al., 2011) and the D-fructose 6-phosphate aldolase (Fsa; Castillo et al., 2006). Based on the mechanism of known aldol reactions, four stereo configurations can be obtained by different specific aldolases (Figure 2A; Samland and Sprenger, 2006). For example, when GALD serves as donor and D-glyceraldehyde 3-phosphate (G3P) serves as acceptor, four isomeric products can be generated (Figure 2B), namely D-lyxose 5-phosphate (L5P), D-arabinose-5P (Ara5P), D-xylose 5-phosphate (X5P), and D-ribose 5-phosphate (R5P). However, only Ara5P (ID: rat131949) and R5P (ID: rat132073) are included in the ATLAS database. In order to design as many novel methanol assimilation pathways as possible, we proposed 28 potential new aldolase reactions whose aldol products contain no more than six carbons to include all possible stereo configurations (Supplementary Table 1).

### Prediction of FALD Assimilation Pathways

**Figure 3:**
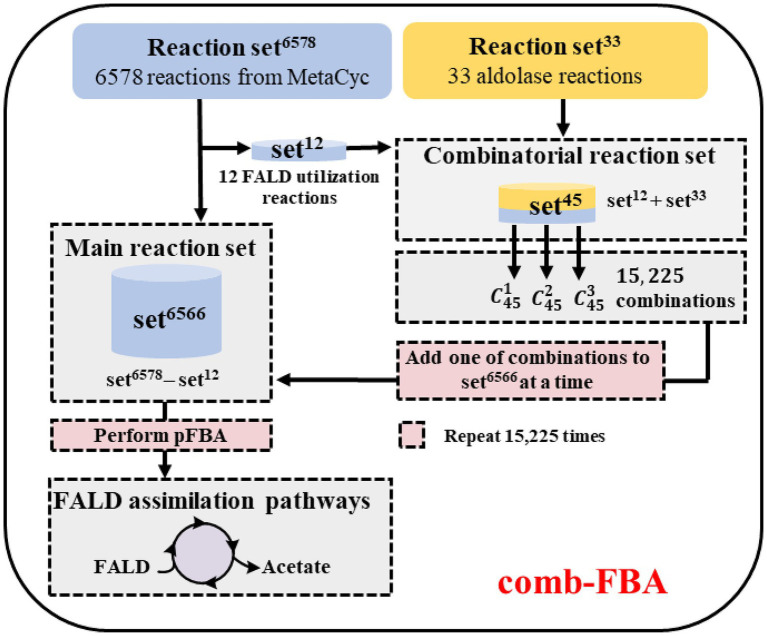
Workflow for the calculation of formaldehyde (FALD) assimilation pathways. Comb-flux balance analysis (FBA) is performed based on two reaction sets: the main reaction set^6566^ and the combinatorial reaction set^45^. In each iteration of comb-FBA calculation, a combination with one reaction ($C_{45}^{1}$), two reactions ($C_{45}^{2}$), or three reactions ($C_{45}^{3}$) from set^45^ was added to the reaction set^6566^ to predict FALD assimilation pathways.

In order to ensure the lowest possible number of non-natural reactions in the predicted pathways, the comb-FBA algorithm (Yang et al., 2019b) was used as shown in Figure 3. The simulated metabolic network contained the known reaction set^6578^ (6,578 MetaCyc reactions) and the aldolase reaction set^33^ (five experimentally verified reactions from ATLAS and 28 artificially proposed reactions; Supplementary Material; Supplementary Table A). Then, 12 known FALD utilization reactions from set^6578^ were extracted together with the aldolase reaction set^33^ to compose the combinatorial reaction set. The remaining set^6566^ (set^6578^ minus set^12^) was taken as the main reaction set. In view of the difficulties in establishing non-natural reactions *in vitro*, we chose no more than three reactions from set^45^, namely 15,225 combinations ($C_{45}^{1}$+$C_{45}^{2}$+$C_{45}^{3})$, to enter the main reaction set^6566^ for subsequent pathway calculation using the pFBA algorithm (Lewis et al., 2010). FALD was set as the substrate, and acetate, the simplest AcCoA derivative, was defined as the objective product.

**Figure 4:**
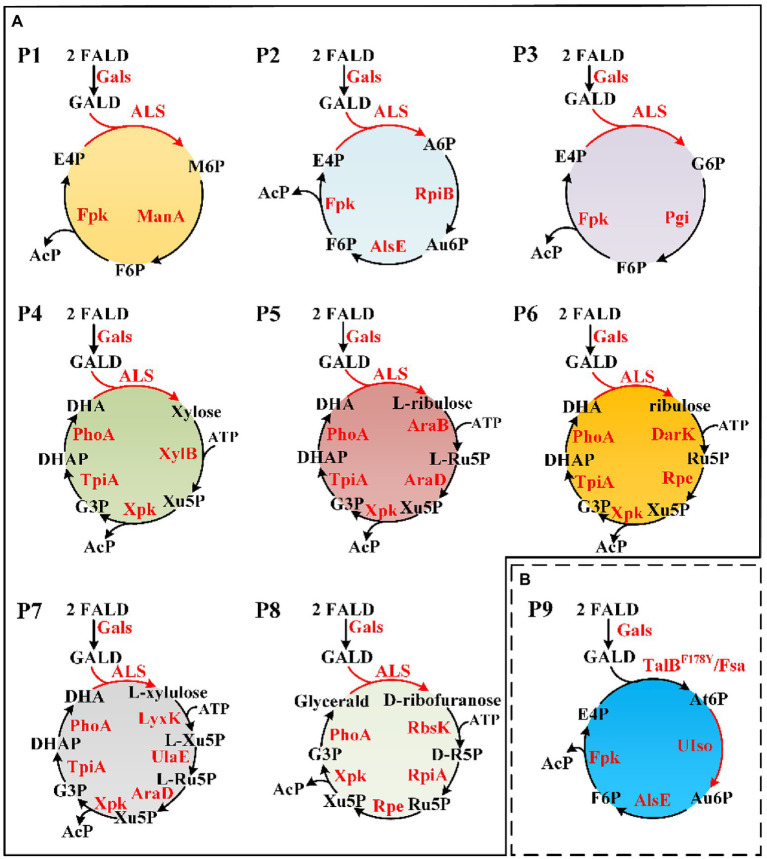
Novel FALD assimilation pathways. **(A)** Predicted pathways (P1–P8). **(B)** Proposed novel pathway P9 based on TalB^Y178F^/Fsa. For simplicity, AcP to Ac (AcP => Acetate) was not shown. M6P, D-mannose 6-phosphate; A6P, D-allose 6-phosphate; Au6P, D-allulose 6-phosphate; G6P, D-glucose 6-phosphate; At6P, D-altrose 6-phosphate; AcP, acetyl-phosphate. Gals, evolved glycolaldehyde synthase; Fpk, fructose 6-phosphate phosphoketolase; ManA, mannose-6-phosphate isomerase; RpiB, allose-6-phosphate isomerase/ribose-5-phosphate isomerase B; AlsE, D-allulose-6-phosphate 3-epimerase; Pgi, glucose-6-phosphate isomerase; XylB, xylulokinase; Xpk, xylulose 5-phosphate phosphoketolase; TpiA, triose phosphate isomerase; PhoA, alkaline phosphatase; AraB, ribulokinase; AraD, L-ribulose-5-phosphate 4-epimerase; DarK, D-ribulokinase; Rpe, ribulose-phosphate 3-epimerase; LyxK, L-xylulose kinase; UlaE, L-ribulose-5-phosphate 3-epimerase; RbsK, ribokinase; RpiA, ribose-5-phosphate isomerase A; TalB, transaldolase; Fsa, fructose 6-phosphate aldolase; and UIso, unknown altrose 6-phosphate isomerase.

Novel FALD assimilation pathways were selected based on the following criteria: (i) no more than 10 reactions from FALD to acetate; (ii) no carbon loss; and (iii) independent of ATP and reducing equivalents. Eight novel FALD assimilation pathways (P1–P8) meeting criteria (i) and (ii) were predicted (Figure 4A). Among them, the pathways P1, P2, and P3, meeting all these criteria, were chosen for further *in vitro* validation.

### Identification of Novel Aldol Reactions for the Condensation of GALD With E4P

Formaldehyde assimilation proceeded through common/similar steps in pathways P1–P3 (Figure 4A). These included the conversion of FALD into GALD by Gals, the condensation of GALD with E4P by unknown ALS enzymes, the isomerization of generated aldohexose 6-phosphates (M6P, A6P, or G6P) to ketohexose 6-phosphates (Au6P and/or F6P) by isomerases and/or epimerases, and the hydrolysis of F6P into E4P and AcP by Fpk. Since the conversion of FALD into GALD has been proven in our previous work (Lu et al., 2019; Yang et al., 2019b), the first cornerstone of constructing pathways P1–P3 was realizing the artificially proposed condensation of GALD with E4P.

**Figure 5:**
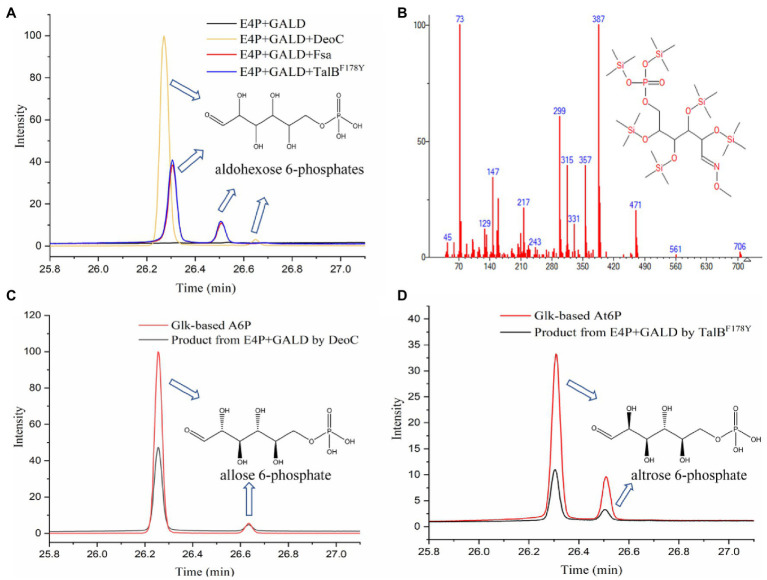
Identification of aldol reaction products by GC-TOFMS. **(A)** The products of the condensation of GALD with E4P by TalB^F178Y^, Fsa, and DeoC. **(B)** Fragment distributions of peaks with retention times of 26.307, 26.274, 26.511, and 26.650 min. **(C)** Glk-based A6P and the product from E4P and GALD catalyzed by DeoC. **(D)** Glk-based At6P and product from E4P and GALD catalyzed by TalB^F178Y^.

Previously, three aldolases with broad donor spectra (TalB^F178Y^, Fsa, and DeoC) were tested for the similar condensation of GALD with G3P, and transaldolase TalB^F178Y^ exhibited the highest activity (Yang et al., 2019b). Therefore, TalB^F178Y^, Fsa, and DeoC (Supplementary Figure 1) were firstly chosen to test if any can catalyze the proposed condensation reaction. The aldol products from the GALD and E4P catalyzed by different aldolases (TalB^F178Y^, Fsa, or DeoC) were derivatized using methoxymation and trimethylsilylation methods (Mairinger et al., 2020), by which the ketone or aldehyde carbonyl groups were converted into methoxyamine groups and the active hydrogen atoms of the hydroxyl groups were replaced by trimethylsilyl groups (Supplementary Figure 2). It is worth mentioning that the methoxymation method produces two different stereoisomers, with either the *syn-* or the *anti-*form of the methoxyamine group (Gullberg et al., 2004; Engel et al., 2020). As shown in Figure 5A, the aldol products of aldolase-catalyzed condensation of GALD with E4P formed two main peaks at 26.307 and 26.511 min (by TalB^F178Y^ or Fsa) or 26.274 and 26.650 min (by DeoC). All the peaks showed similar fragment distributions (Figure 5B), which were consistent with aldohexose 6-phosphates according to the Agilent NIST2020 GC-TOFMS libraries. However, standards or equivalents were required to distinguish the number and type of stereoisomers among the aldol reaction products.

As we failed to obtain an A6P standard, an alternative method, using Glk to produce aldohexose 6-phosphates from corresponding aldohexoses, was tried. The reaction products from different aldohexoses (D-glucose, D-mannose, or D-allose) catalyzed by Glk were qualitatively analyzed using the same GC-TOFMS method. As shown in Supplementary Figure 3, three aldohexose 6-phosphates (G6P, M6P, and A6P) were successfully produced by Glk from D-glucose, D-mannose, and D-allose, respectively. The aldolase product of DeoC was identified as A6P, since its GC-TOFMS retention time was identical with that of Glk-based A6P (Figure 5C). However, the aldohexose 6-phosphate generated by TalB^F178Y^/Fsa remained unknown, as its retention time was not consistent with any of G6P, M6P, and A6P.

After checking the possible aldol reaction products (Figure 2; Supplementary Table 1), altrose 6-phosphate (At6P), which was previously omitted as it was not included in the MetaCyc database, was identified as a likely candidate for the missing stereoisomer. Indeed, the retention time of the unknown aldohexose 6-phosphate was consistent with that of Glk-based altrose 6-phosphate (Figure 5D). Therefore, the aldol product of TalB^F178Y^/Fsa was identified as At6P, and the pathway P9 with non-natural isomerization of At6P into Au6P was newly proposed (Figure 4B). Thus, we finally identified two novel aldol reactions for the condensation of GALD with E4P into 2*S*,3*R*-configurated At6P by TalB^F178Y^/Fsa or 2*R*,3*R*-configurated A6P by DeoC.

### Isomerization of A6P Into F6P by RpiB and AlsE

**Figure 6:**
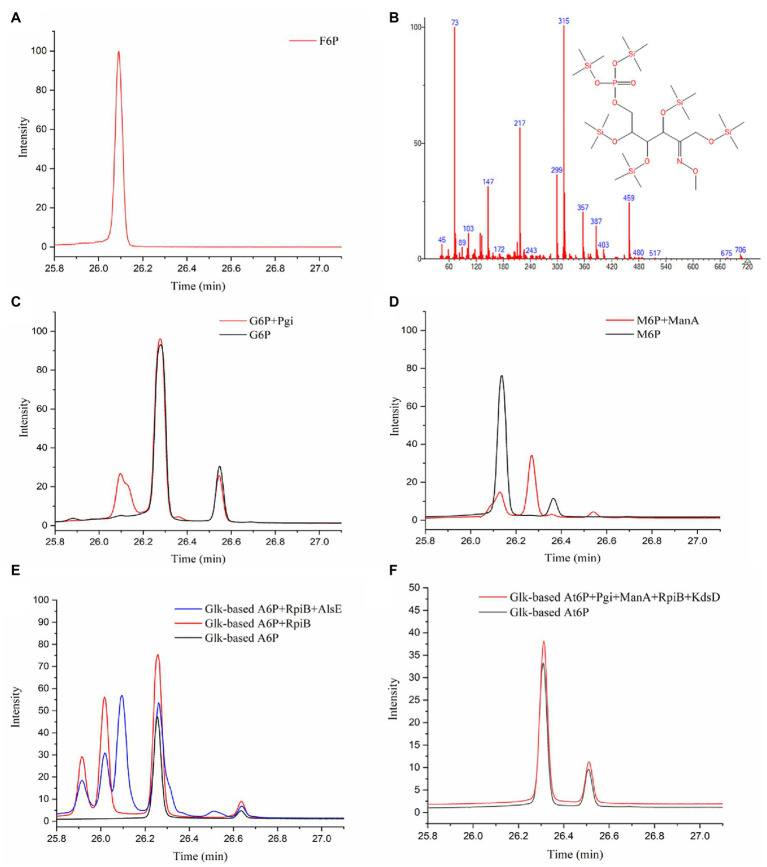
Identification of isomerized products from aldohexose 6-phosphates by GC-TOFMS method. **(A)** F6P standard. **(B)** Fragment distributions of ketohexose 6-phosphates produced *via* the isomerization of G6P, M6P, or A6P with retention times of 25.915, 26.018, and 26.094 min. **(C)** G6P standard and product from G6P catalyzed by Pgi. **(D)** M6P standard and product from M6P catalyzed by ManA. **(E)** Glk-based A6P and product from A6P catalyzed by RpiB (and AlsE). **(F)** Glk-based At6P and product from At6P catalyzed by Pgi, ManA, RpiB, and KdsD.

The next step following the condensation of GALD with E4P was predicted to be the isomerization of the obtained aldohexose 6-phosphate into F6P. The reaction products from different aldohexose 6-phosphates catalyzed by different isomerases (Pgi, ManA, RpiB, KdsD, and/or AlsE) were qualitatively analyzed using GC-TOFMS. According to the generated peaks and their retention time, Glk-based G6P and M6P were successfully isomerized into F6P by Pgi and ManA, respectively (Figures 6A–D). A6P was isomerized into another ketohexose 6-phosphate by RpiB, followed by epimerized to F6P by AlsE (Figure 6E). Although, we failed to obtain an Au6P standard or equivalent, this ketohexose 6-phosphate was probably Au6P because (1), the 3*R*-configurated A6P/At6P was supposed to be isomerized into 3*R*-configurated Au6P rather than 3*S*-configurated F6P (Supplementary Figure 4), and (2), RpiB was reported to be able to isomerize A6P into Au6P (Roos et al., 2008), while Au6P could be epimerized into F6P by AlsE as described by Chan et al. (2008). Thus, enzymes catalyzing all the reactions in pathway P2 were identified.

Because At6P is an unnatural metabolite and was not included in the MetaCyc database, no known isomerization reaction for At6P was available. Therefore, we examined three aldohexose 6-phosphate isomerases (RpiB, ManA, and Pgi) as well as the arabinose-5-phosphate isomerase (KdsD) to see if any could isomerize At6P into Au6P. Unfortunately, no ketohexose 6-phosphate was observed in the systems containing Glk-based At6P with all the aldose phosphate isomerases (Figure 6F), resulting in a temporary failure to realize pathway P9 (Figure 4B). Nevertheless, these isomerases did not show strict stereo selectivity for G6P, M6P, or A6P (Supplementary Figures 3H–J). This result suggested that 2*S*,3*R*-At6P prefers specific aldohexose 6-phosphate isomerases.

### *In vitro* Construction of the GAPA Pathway

**Figure 7:**
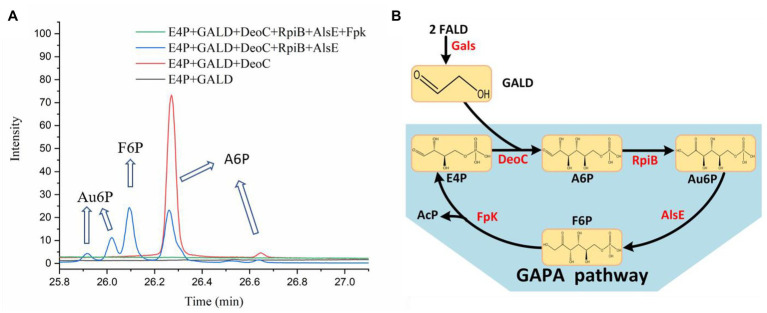
Identification of the GAPA pathway. **(A)** The reaction products of the condensation of GALD with E4P by DeoC, RpiB, AlsE, and/or Fpk as determined by GC-TOFMS. **(B)** The proved GAPA pathway.

**Figure 8:**
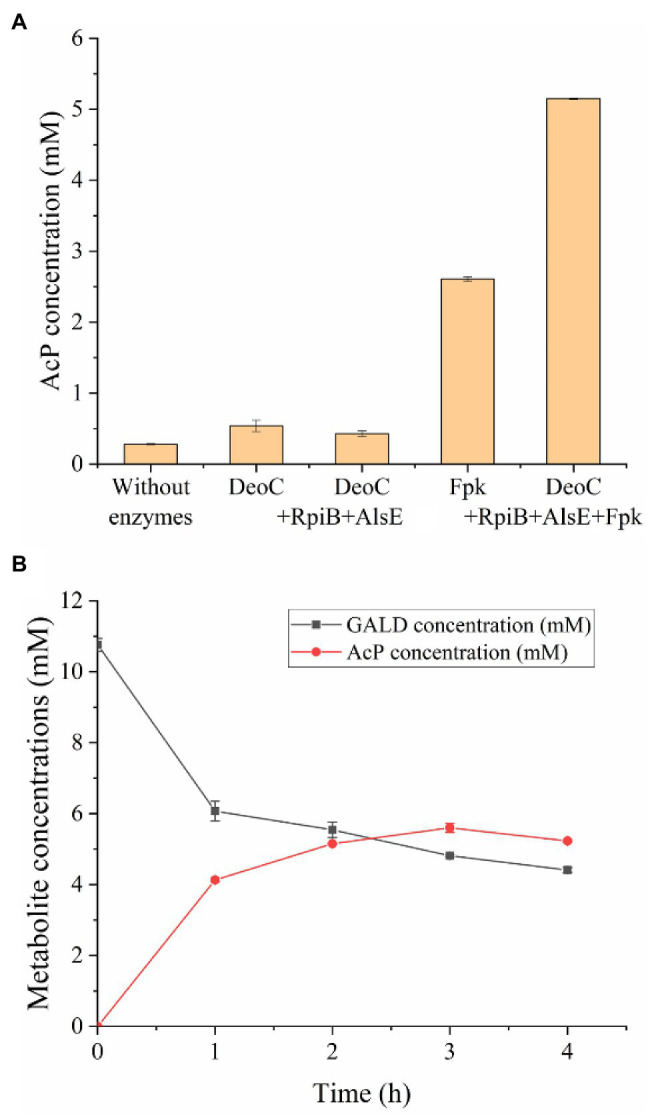
Efficiency evaluation of the GAPA pathway. **(A)** AcP concentrations produced from 2.5 mM E4P and 10 mM GALD after 2 h using different enzymes (DeoC 2 mg/ml, RpiB 2 mg/ml, AlsE 2 mg/ml, and/or Fpk 1 mg/ml). **(B)** Time profile of GALD and AcP concentration in reaction system containing 10 mM GALD, 2.5 mM E4P, 2 mg/ml DeoC, 2 mg/ml RpiB, 2 mg/ml AlsE, and 1 mg/ml Fpk.

In order to test the feasibility of the GAPA pathway, we assembled the purified enzymes *in vitro* (DeoC, RpiB, AlsE, and/or Fpk) with GALD and E4P as reaction substrates. As shown in Figure 7A, when DeoC, RpiB, and AlsE were successively added into the reaction system, GALD and E4P were condensed into A6P, then isomerized to Au6P, and finally epimerized into F6P. After further addition of Fpk, all the peaks for ketohexose 6-phosphate or aldohexose 6-phosphate significantly decreased, which indicated that the GAPA pathway (Figure 7B) was successfully constructed. Subsequently, the concentrations of AcP generated by the different reaction systems were determined to evaluate the efficiency of the GAPA pathway. As shown in Figure 8A, very little AcP could be obtained without the addition of Fpk, which might be caused by unclearly spontaneous reaction. When only Fpk was added, 2.61 mM AcP was obtained after 2 h, likely representing the reported direct conversion of GALD into AcP by phosphoketolase (Lu et al., 2019). However, when all the enzymes were added into reaction system (DeoC 2 mg/ml, RpiB 2 mg/ml, AlsE 2 mg/ml, and Fpk 1 mg/ml), the AcP concentration was significantly increased by 97% to 5.15 mM after 2 h (Figure 8A), indicating that the GAPA pathway did play a major role in AcP synthesis. After 3 h, the maximal AcP concentration of 5.60 mM was obtained, while 5.95 mM GALD was consumed (Figure 8B), corresponding to a carbon yield of 94% for GALD.

## Discussion

Artificial construction of non-natural reactions has facilitated the *de novo* design of metabolic pathways (Kumar et al., 2018; Saa et al., 2019; Hafner et al., 2020), among which the screening of new aldolase reactions plays an important role in mining novel one-carbon assimilation pathways (Siegel et al., 2015; Lu et al., 2019; Yang et al., 2019b; He et al., 2020). In this study, we identified 28 novel theoretically feasible, non-natural aldolase reactions, and based on these, calculated eight simple, novel, and carbon-conserving FALD assimilation pathways (P1–P8, Figure 4A) using the comb-FBA algorithm (Figure 2). Notably, three of the predicted FALD assimilation pathways (P1–P3), proceeding *via* GALD and different aldohexose 6-phosphates, were independent from energy/reducing equivalents seen from FALD to AcP, and were therefore given with priority for experimental verification. Since the feasibility for their common conversion of FALD into GALD by Gals has been proven (Lu et al., 2019; Yang et al., 2019b), this work focused on the identification of unknown aldolase reactions and the *in vitro* construction of pathways P1–P3 starting from GALD.

During the condensation of GALD with E4P, two new asymmetric centers are formed, resulting in four theoretical stereo configurations (2*R*,3*S*-G6P, 2*S*,3*S*-M6P, 2*R*,3*R*-A6P, and 2*S*,3*R*-At6P). Although specific dihydroxyacetone phosphate (DHAP) aldolases are able to generate corresponding stereo configurations (Samland and Sprenger, 2006; Clapés et al., 2010), DHAP aldolases are usually strictly specific for DHAP as the donor (Gefflaut et al., 1995). Therefore, two well-known DHA aldolases with tolerances for different aldol donors, TalB^F178Y^ and Fsa (Garrabou et al., 2009; Rale et al., 2011; Lachaux et al., 2019), were tried and successfully condensed GALD with E4P into the 2*S*,3*R*-stereo configuration At6P (Figure 5D). This 2*S*,3*R*-configuration was consistent with the strict stereoselectivity of DHA aldolases for the *S*,*R*-stereo configuration during aldol reactions (Clapés et al., 2010). However, aldolase candidates for the other three configurations were rarely reported.

Interestingly, the 2-deoxy-D-ribose-5-phosphate aldolase (DERA, encoded by *deoC* in *E. coli*) exhibited a different stereoselectivity for the 2*R*,3*R*-stereo configuration (Figure 5C). In our previous work, the product of GALD and G3P catalyzed by DeoC exhibited similar retention times with those by TalB^F178Y^/Fsa during GC-TOFMS analysis (Yang et al., 2019b). These were supposed to be the 2*S*,3*R*-stereo arabinose 5-phosphate (Ara5P) according to their acceptance as substrates by known isomerases. In this study, a simple but more rigorous method was adopted to qualitatively analyze the aldol products. The equivalents of G6P, M6P, A6P, and At6P were produced by Glk from corresponding aldohexoses (Supplementary Figure 3). Subsequently, GC-TOFMS analysis coupled with isomerization by known aldose phosphate isomerases was used to judge the stereo configuration of the aldol products. Thus, the aldol product of GALD and E4P by DeoC was determined as the 2*R*,3*R*-form A6P. Chambre et al. (2019) recently discovered that the DERA from *Arthrobacter chlorophenolicus*, sharing a 34% amino acid sequence identity with DeoC from *E. coli*, also exhibited selectivity for the *R*,*R*-stereo configuration when using GALD or DHA as donors and L-glyceraldehyde 3-phosphate as acceptor. It is worth mentioning that the identification of both the non-natural production of At6P production from GALD and E4P by TalB^F178Y^/Fsa and the rare *R*,*R*-stereo configuration aldolase reaction catalyzed by DeoC may facilitate future biosynthesis of high-valued rare saccharides in various stereoisomers (Roca et al., 2015; Yang et al., 2019a; Li et al., 2021). However, the underlying mechanism of the different stereoselectivity between DHA aldolase and DERA need further investigation.

After the aldol products were clearly distinguished as 2*S*,3*R*-form At6P or 2*R*,3*R*-form A6P, efforts were paid to isomerize them into F6P. After A6P was successfully converted into F6P by RpiB and AlsE, the DeoC-based GAPA pathway was finally constructed *in vitro*, exhibiting a high carbon yield of 94% from GALD (Figure 8B), which confirmed the feasibility of mining novel pathways using model-based pathway design combined with artificially proposed reactions. Moreover, it is worth noting that more novel one-carbon assimilation pathways could be identified with future efforts to realize aldol condensation for 2*S*,3*S*-stereo M6P or 2*R*,3*S*-stereo G6P (pathways P1 and P3, Figure 4A), as well as the isomerization of At6P into Au6P (pathway P9, Figure 4B). We believe that the GAPA pathway is a promising candidate for a GALD-based one-carbon assimilation pathway if it is coupled with a kinetically favorable bioconversion of FALD into GALD using either Hacl and Acr (Chou et al., 2019) or an improved variant of Gals (Lu et al., 2019). The GAPA pathway still faced many other problems such as kinetic trap caused by broad substrate activities of Fpk (Yang et al., 2019b). However, promiscuous enzymes with side reactions in addition to the main reactions are universal *in vivo* and there are many metabolic engineering studies using promiscuous enzymes such as aldolases and PKTs without any problem (Yang et al., 2019a; Hellgren et al., 2020). Moreover, since the pathway discovery and enzyme engineering are mutually reinforcing each other, we believe that the promiscuities and kinetics problems of used enzymes can be eventually overcame by further development of enzyme engineering. Therefore, the GAPA will be feasible *in vivo* with future efforts. Besides one-carbon compound assimilation, these GALD-based pathways also provide promising alternative routes for assimilation of GALD generated from the poly(ethylene glycol) plastics, which is one of the most widely used biopolymers in the pharmaceutical industry (Knop et al., 2010). Taken together, this work not only provides an elegant paradigm for systematic pathway mining, but also uncovers non-natural aldolase reactions with rarely reported product stereoselectivity, which offers novel elements for valuable biosynthesis/biodegradation processes more than one-carbon assimilation.

## Data Availability Statement

The original contributions presented in the study are included in the article/Supplementary Material, further inquiries can be directed to the corresponding author.

## Author Contributions

QY and YM designed pathway. YM, XY, YC, and JL performed the experiments. YY and HS prepared the samples. YM, XY, JL, HL, TC, PL, and HM performed the data analysis. YM, QY, XY, and HM wrote and revised the manuscript. HM, YM, and QY conceived the concept. HM supervised the work. All authors contributed to the article and approved the submitted version.

### Conflict of Interest

The authors declare that the research was conducted in the absence of any commercial or financial relationships that could be construed as a potential conflict of interest.
