# Supplementary material for: Non-natural Aldol Reactions Enable the Design and Construction of Novel One-Carbon Assimilation Pathways in vitro
Source: Front Microbiol. 2021 Jun 2;12:677596. doi: 10.3389/fmicb.2021.677596 (PMC8208507; doi:10.3389/fmicb.2021.677596)
Supplement: Supplementary file 2 [file Image_1.PDF]

Supplementary Material

Supplementary Figures

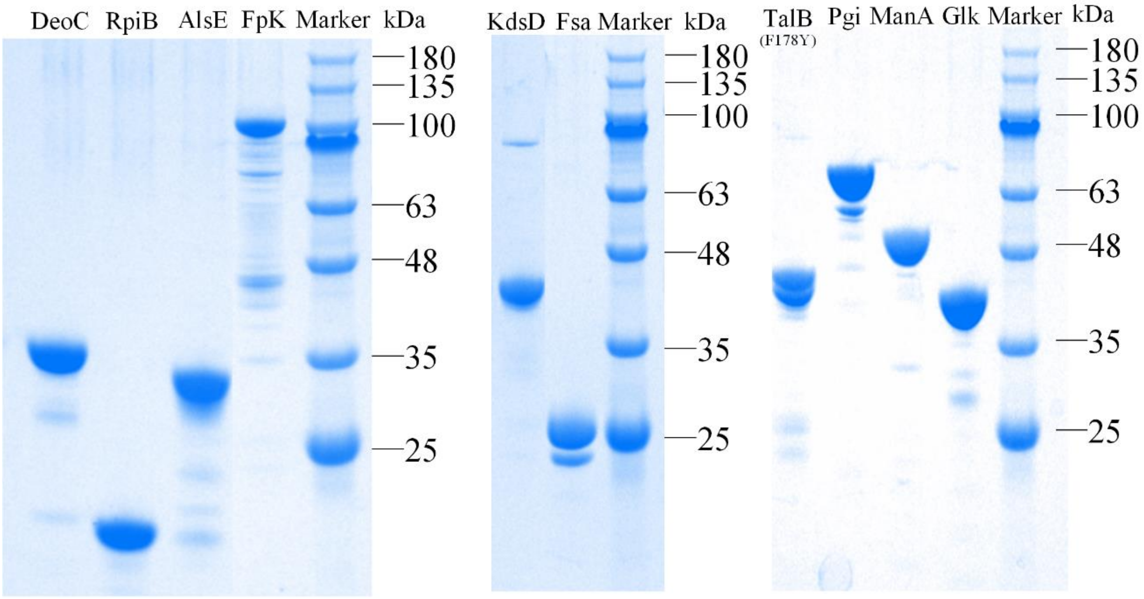

Figure 1 SDS-PAGE analysis of purified enzymes used in this study. Expected molecular masses of these His-tagged proteins: DeoC, ~31 kDa; RpiB, ~19 kDa; AlsE, ~29 kDa; Fpk, ~96 kDa; KdsD, ~39 kDa; Fsa, ~26 kDa; $TalB^{F178Y}$, ~37 kDa; Pgi, ~66 kDa; ManA, ~46 kDa; Glk, ~39 kDa.

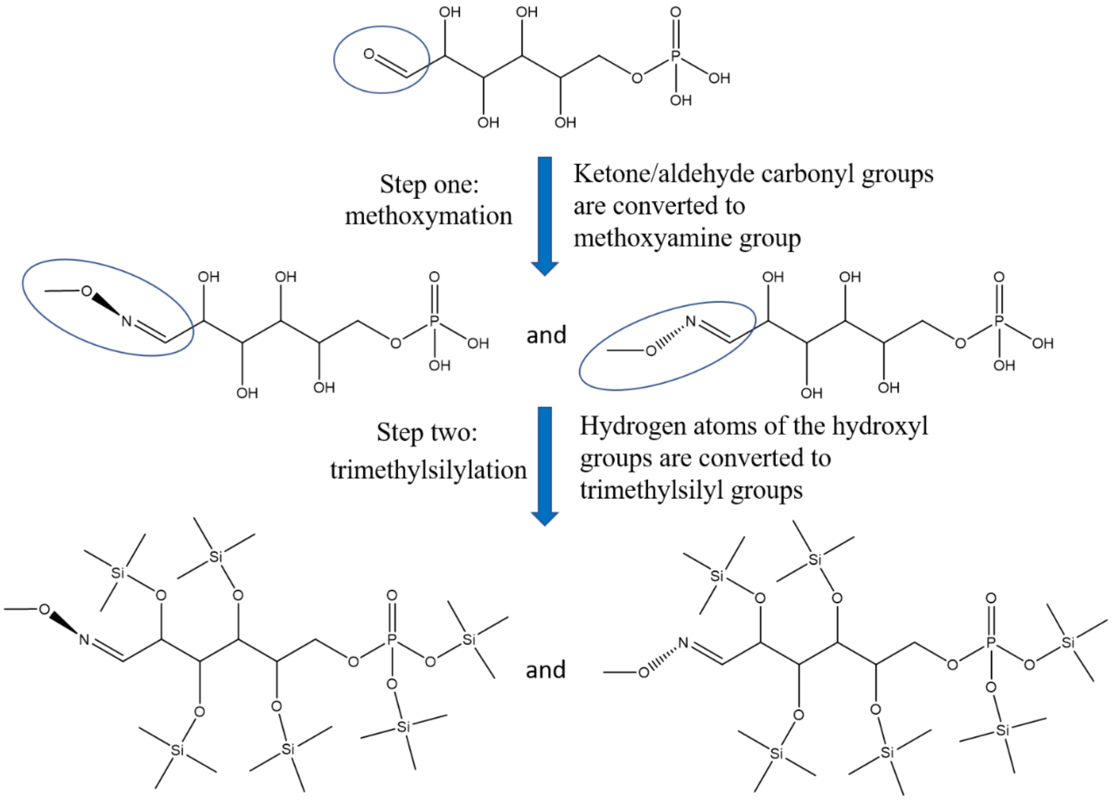

Figure 2 Schematic diagram for two-step derivatization using methoxymation and trimethylsilylation method.

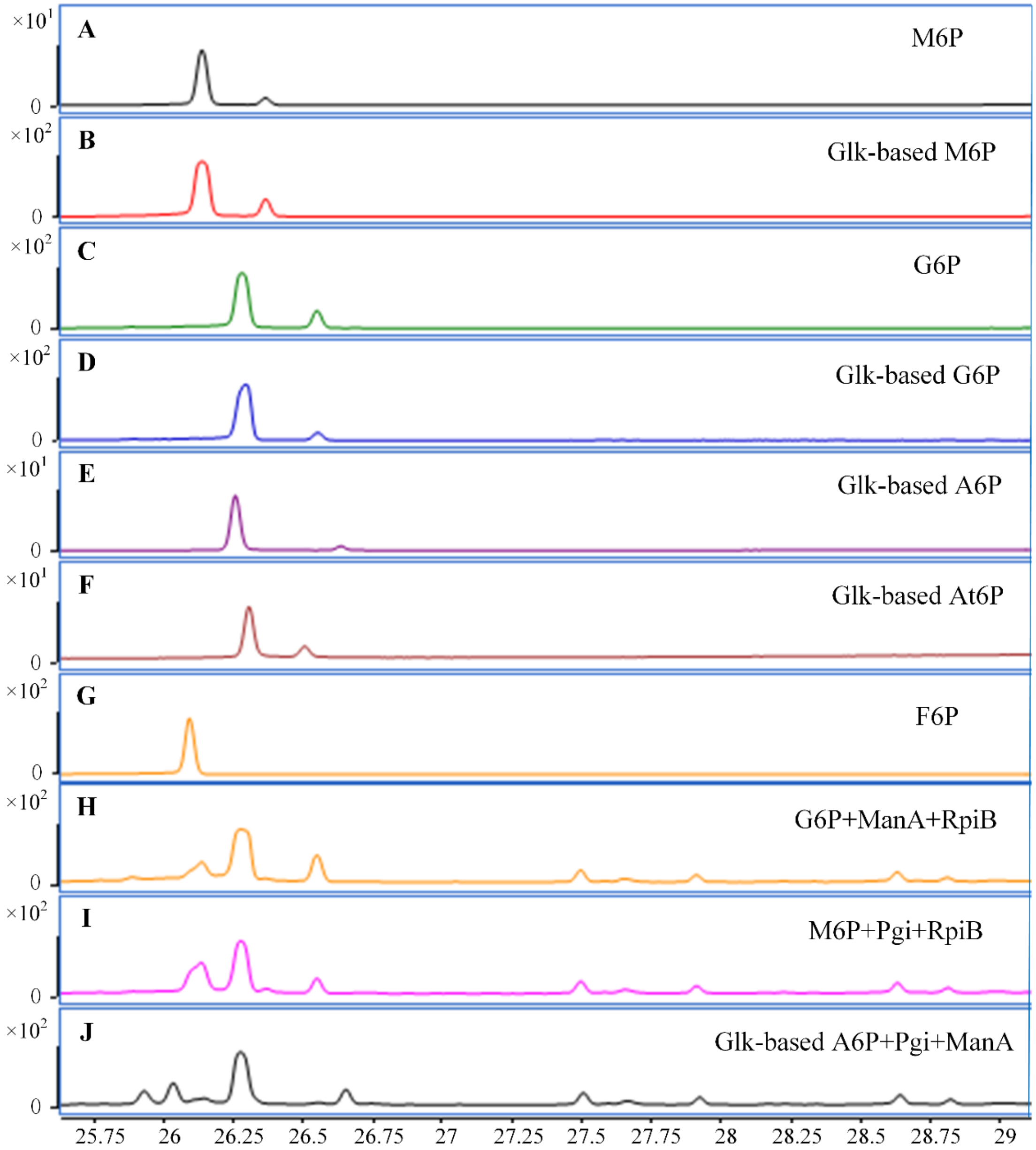

Figure 3 Standards and reaction products as determined by GC-TOFMS. A. M6P standard; **B.** M6P produced from mannose by Glk; **C.** G6P standard; **D.** G6P produced from glucose by Glk; **E.** A6P produced from allose by Glk; **F.** At6P produced from altrose by Glk; **G.** F6P standard; **H.** Isomerized product produced from G6P by ManA and RpiB; **I.** Isomerized product produced from M6P by Pgi and RpiB; **J.** Isomerized product produced from Glk-based A6P by Pgi and ManA.

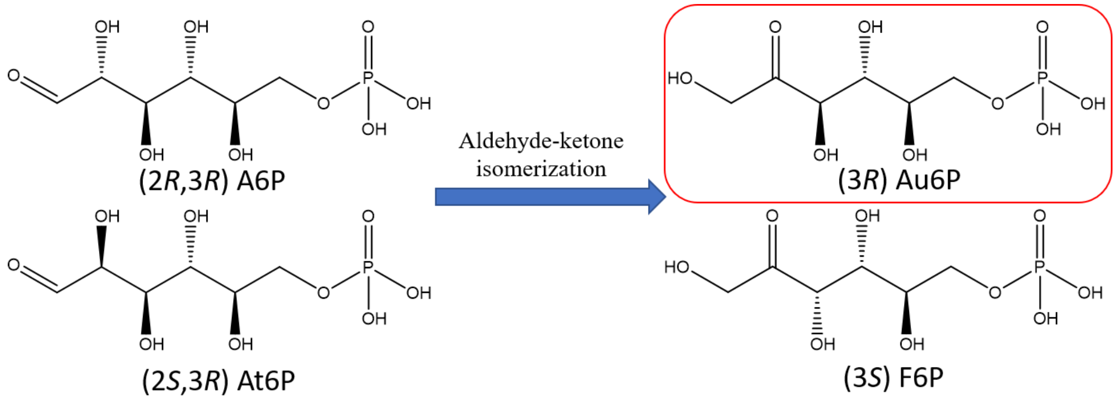

Figure 4 Schematic diagram of the isomerization of 3*R*-A6P/At6P into 3*R*-Au6P.

Supplementary Table

Table 1. Newly proposed aldolase reactions with products present in MetaCyc database (No. 1-28) or not present in MetaCyc database (No. R1-R20).

Characteristic	Reaction ID	Reaction
**FALD as Acceptor:**	**1**	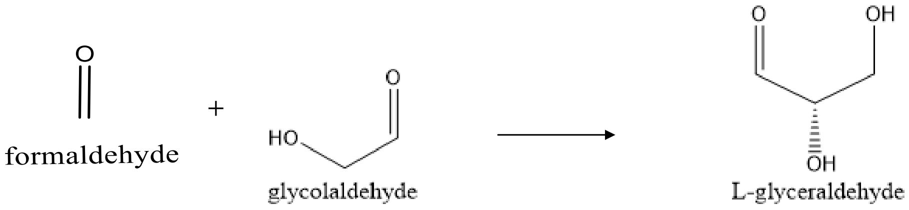
	**2**	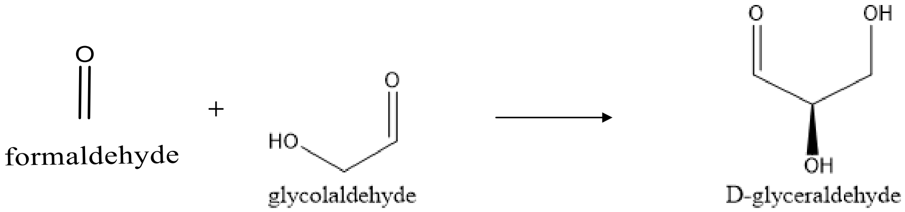
	**3**	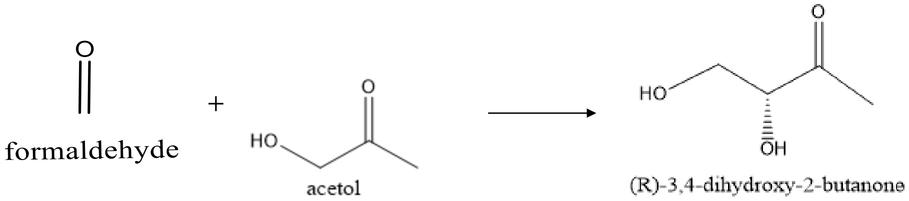
	**R1**	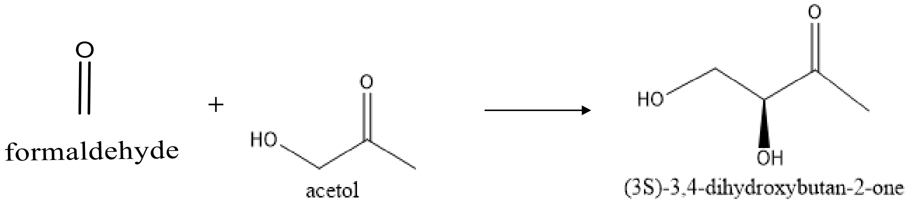
	**4**	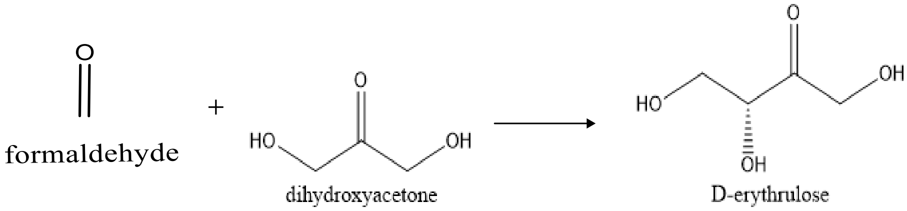

Characteristic	Reaction ID	Reaction
	**R2**	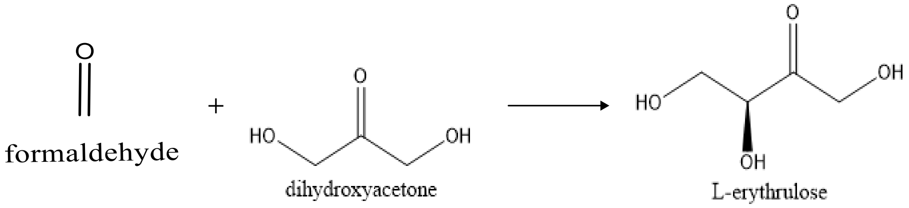
	**5**	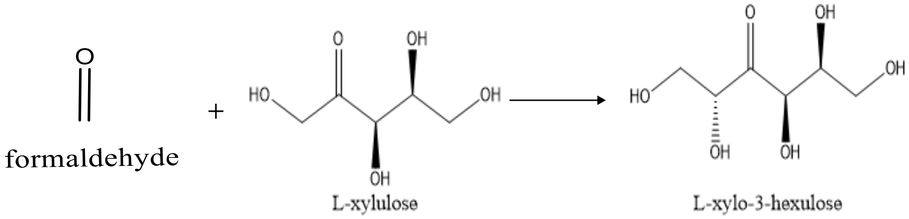
	**R3**	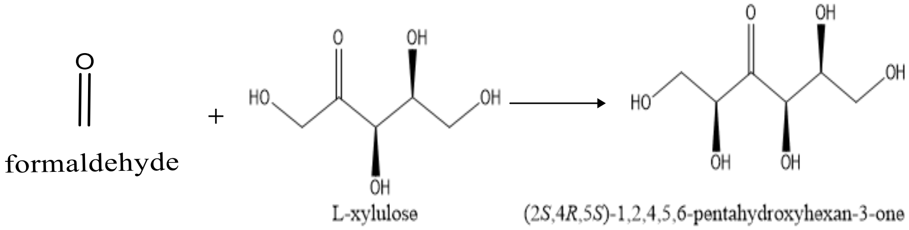
	**R4**	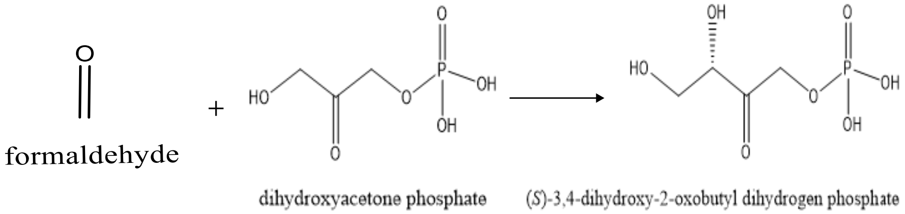
	**R5**	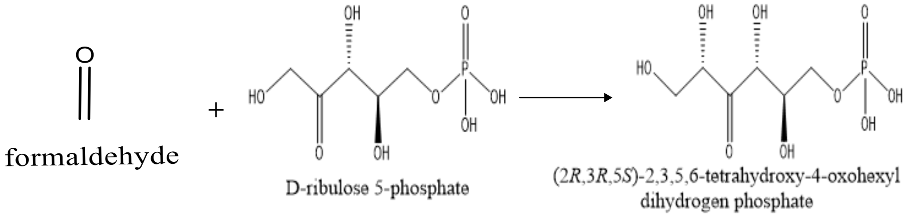

Characteristic	Reaction ID	Reaction
GALD as Acceptor:	6	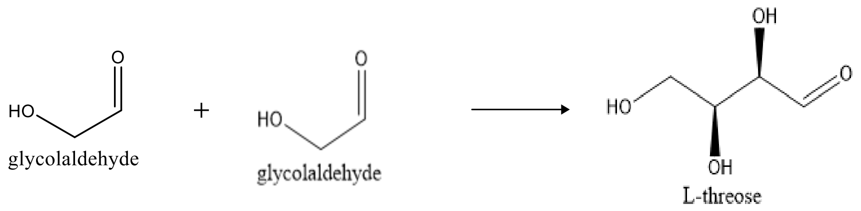
	7	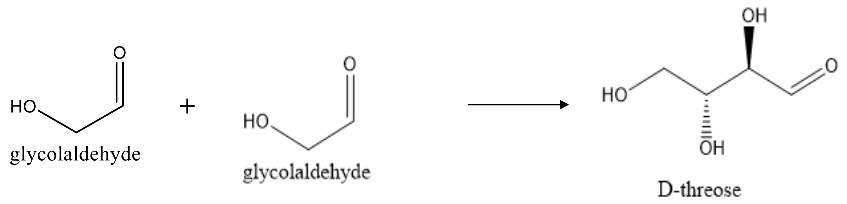
	R6	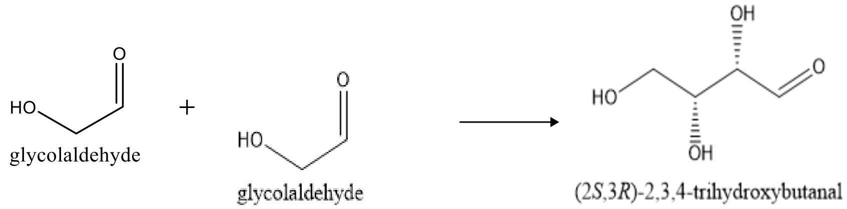
	R7	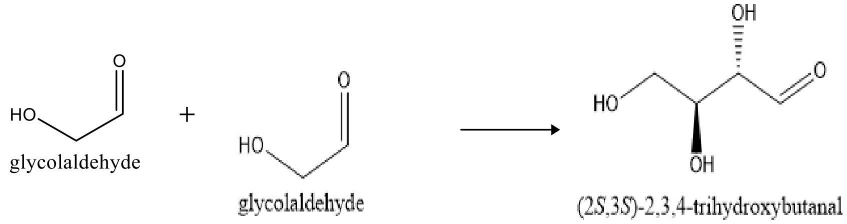
	8	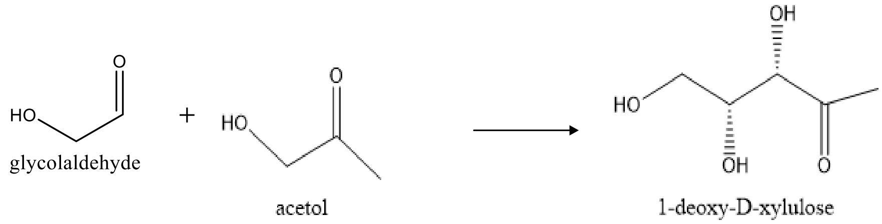
	R8	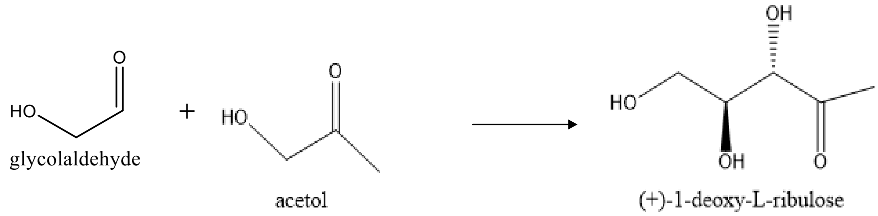

Characteristic	Reaction ID	Reaction
	**R9**	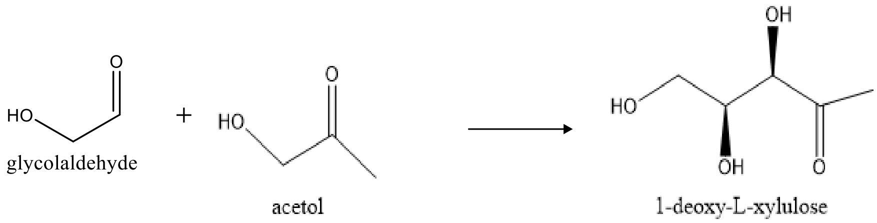
	**R10**	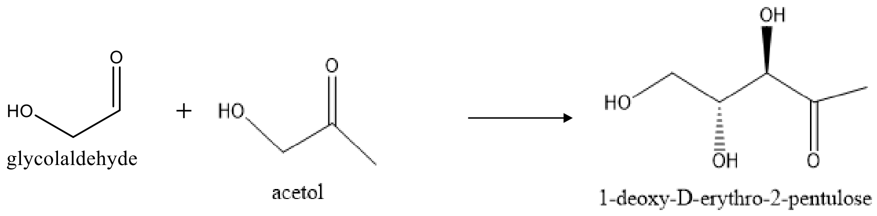
	**9**	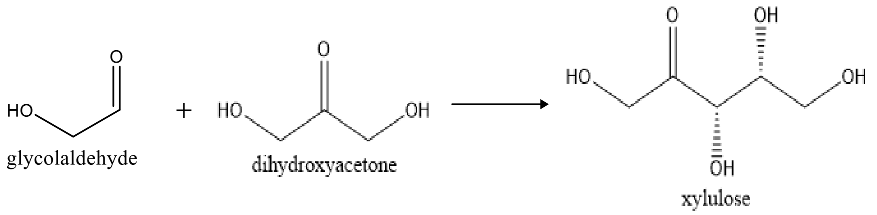
	**10**	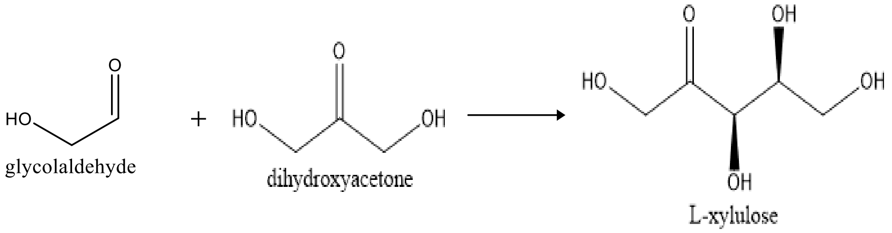
	**11**	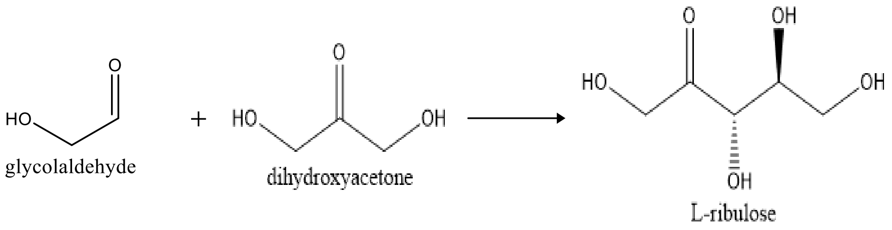

Characteristic	Reaction ID	Reaction
	**12**	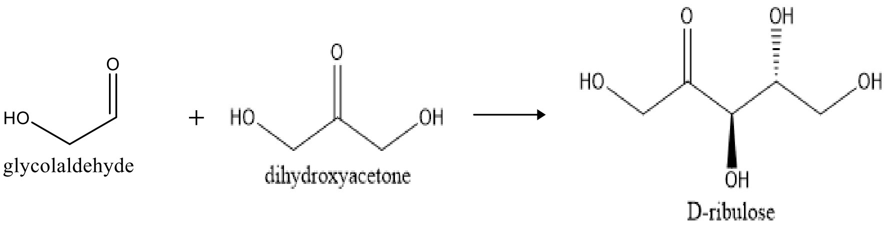
	**13**	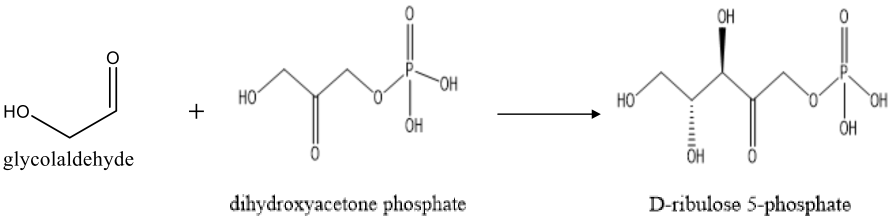
	**14**	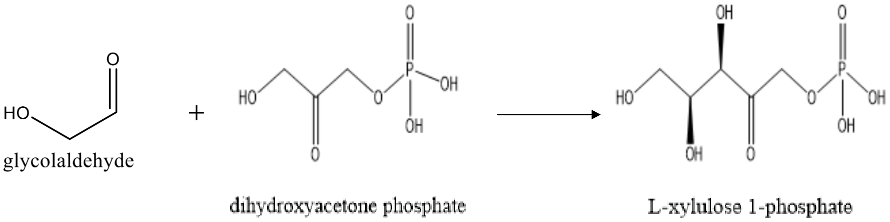
	**R11**	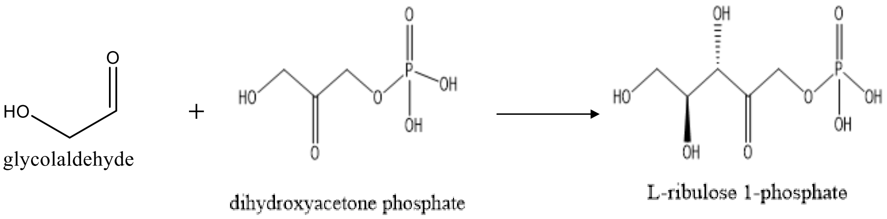
	**R12**	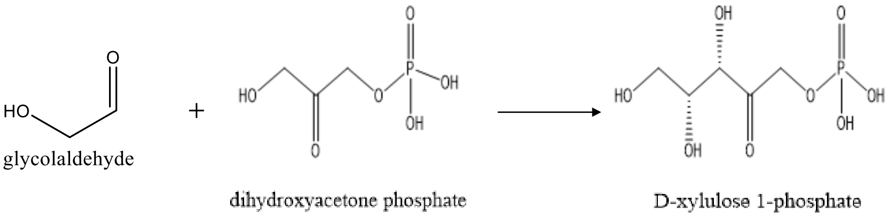

Characteristic	Reaction ID	Reaction
	**15**	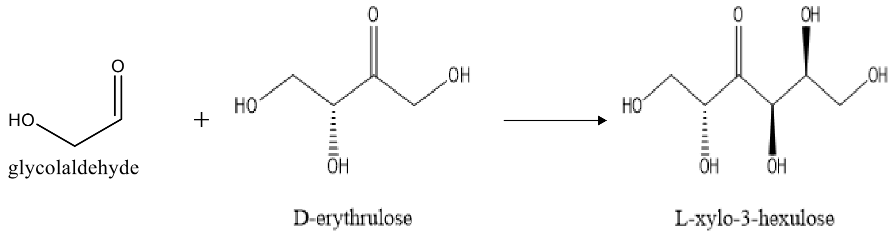
	**R13**	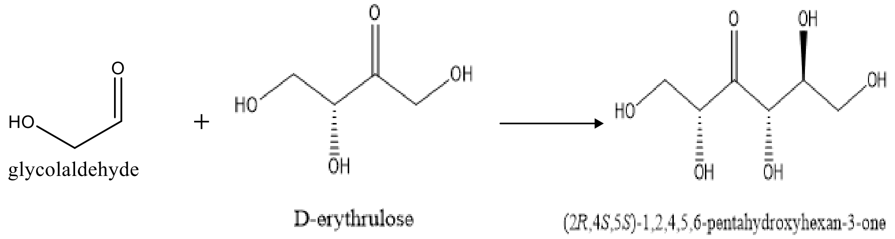
	**R14**	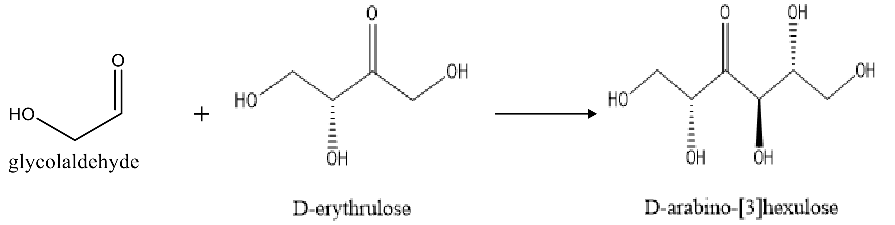
	**R15**	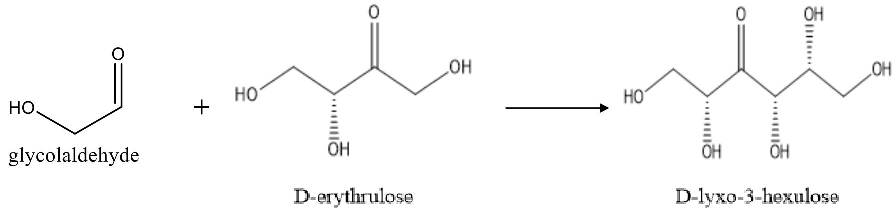
**GALD as Donor**	**16**	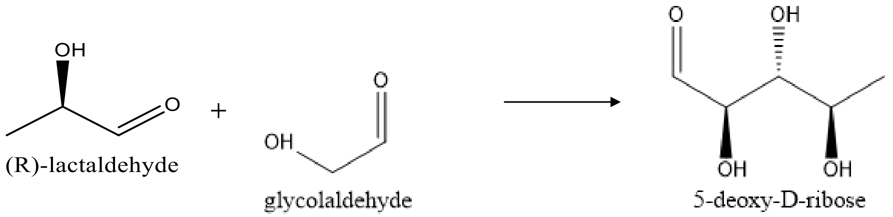

Characteristic	Reaction ID	Reaction
	**R16**	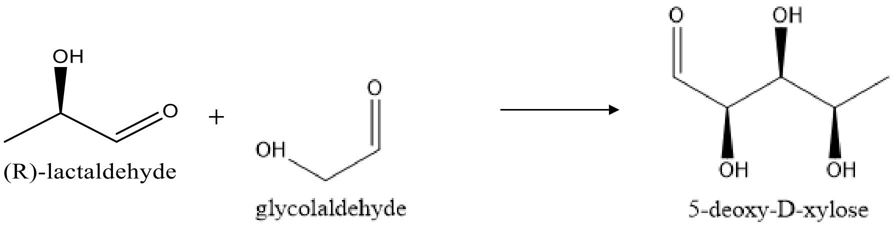
	**R17**	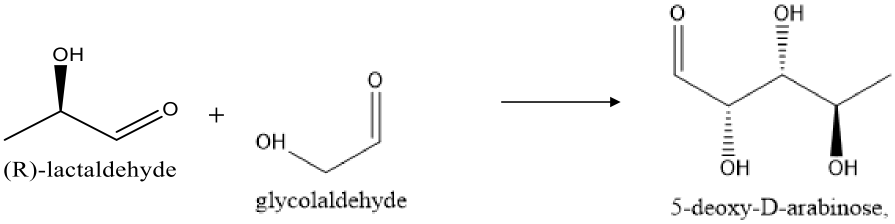
	**R18**	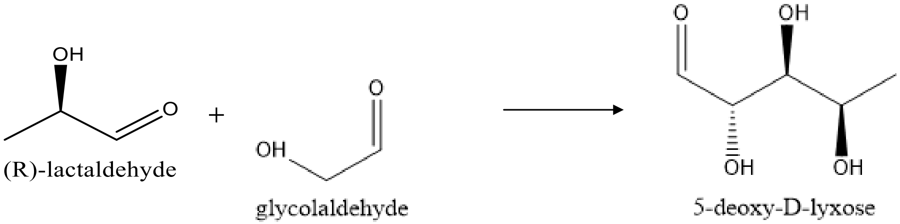
	**17**	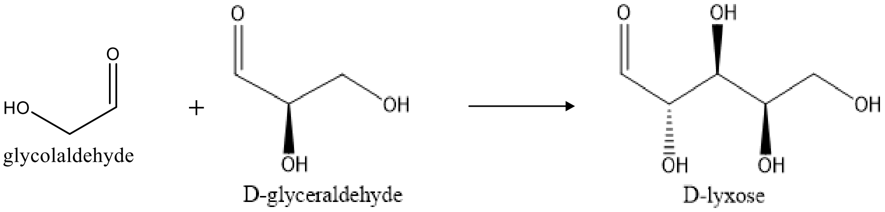
	**18**	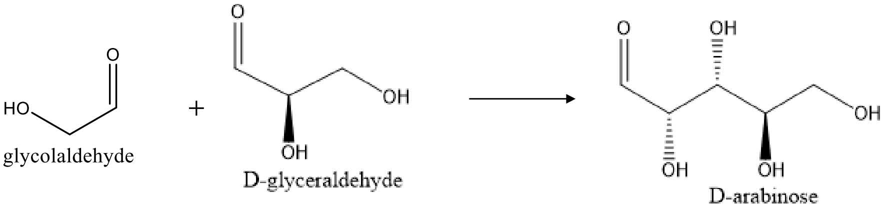
	**19**	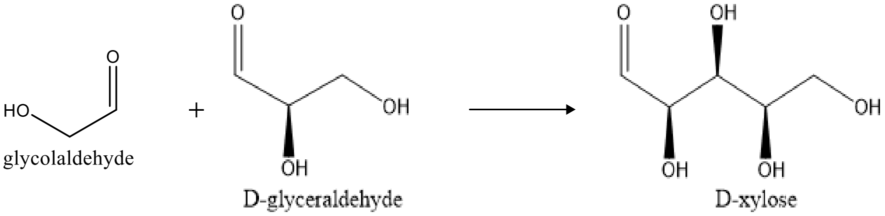

Characteristic	Reaction ID	Reaction
	**20**	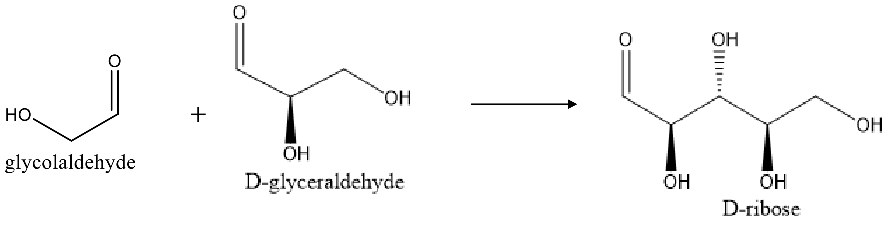
	**21**	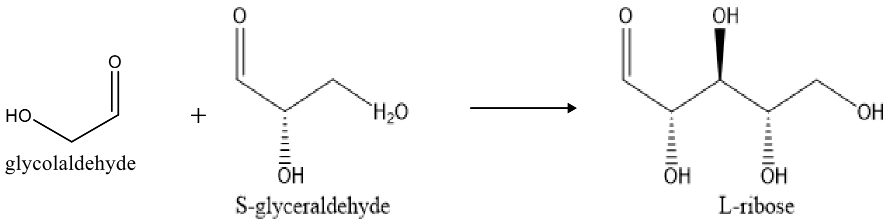
	**22**	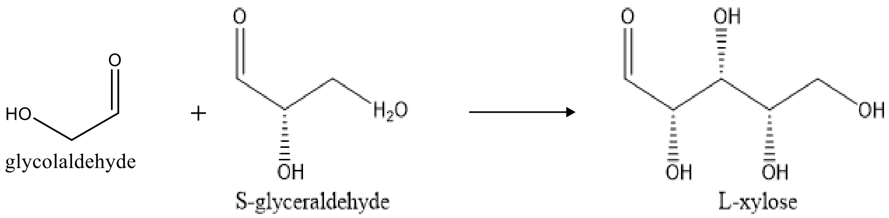
	**23**	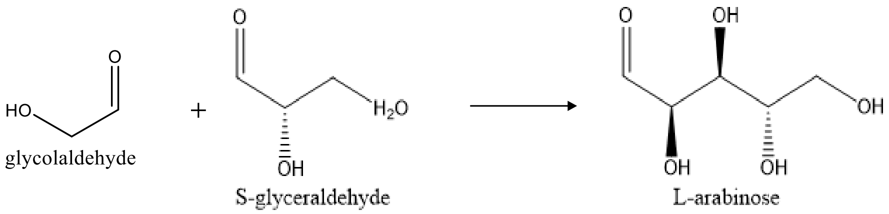
	**24**	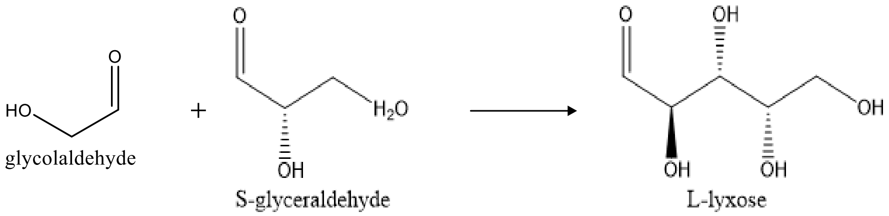

Characteristic	Reaction ID	Reaction
	**25**	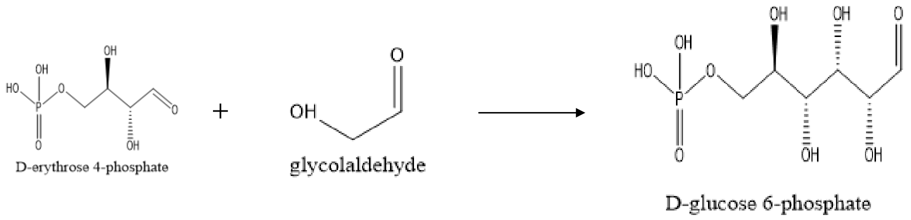
	**26**	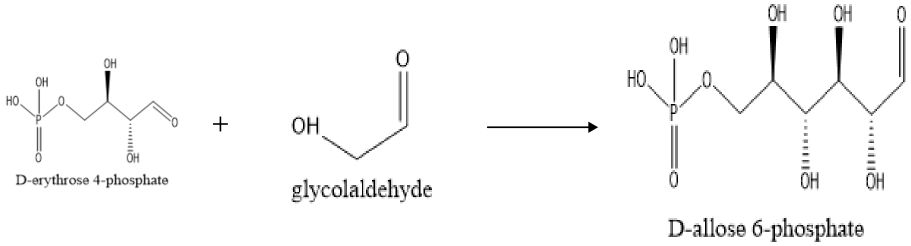
	**27**	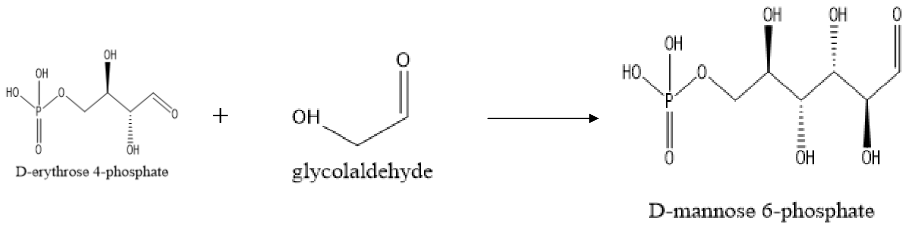
	**R19**	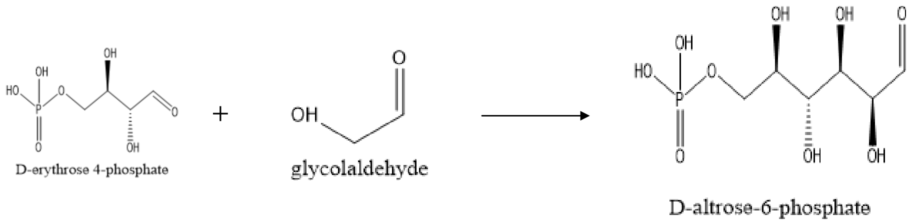
	**28**	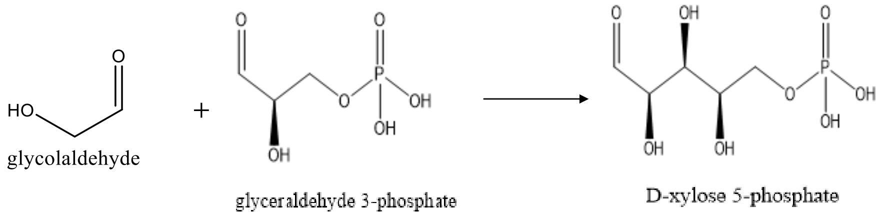

Characteristic	Reaction ID	Reaction
	**R20**	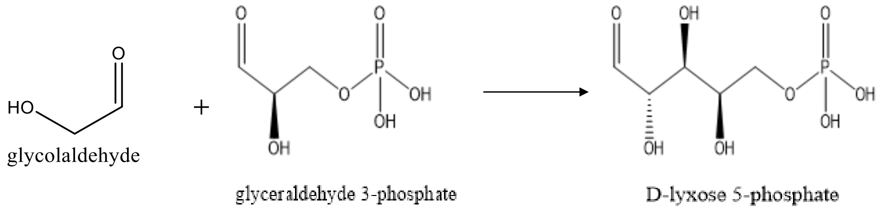
